# Anti-trophoblast Cell-Surface Antigen 2 (TROP2)-Based Therapy for Concurrent Breast and Thyroid Malignancies

**DOI:** 10.7759/cureus.117545

**Published:** 2026-10-06

**Authors:** Maurice J Berkowitz, Karo K Arzoo

**Affiliations:** 1 Hematology and Medical Oncology, University of California Los Angeles David Geffen School of Medicine, Los Angeles, USA

**Keywords:** metastatic breast cancer (mbc), metastatic papillary thyroid cancer, sacituzumab, sacituzumab govitecan, trop2

## Abstract

The simultaneous presence of thyroid carcinoma with other non-thyroid malignancies poses unique treatment dilemmas. Optimal treatment strategies for patients with simultaneous papillary thyroid carcinoma and breast cancer have not been clearly identified. There is increasing attention focused on targeting molecular markers common to both tumors, such as trophoblast cell surface antigen 2 (Trop-2). Herein, we report an 86-year-old female with hormone receptor-positive, HER2-low metastatic breast cancer and an RAI-refractory, BRAF V600E-mutated, metastatic papillary thyroid carcinoma with Trop-2 expression. Following four cycles of sacituzumab govitecan for the breast cancer, she was re-staged. Radiographic studies revealed a partial response to the regimen for the pulmonary metastases along with improvement in thyroglobulin levels and marked reduction of breast tumor markers. This case highlights the possibility of cross-tumor efficacy of Trop-2 targeted agents, thus providing the rationale that sacituzumab govitecan should be explored in RAI-refractory differentiated thyroid cancer.

## Introduction

Papillary thyroid carcinoma is the most common histologic subtype of thyroid carcinoma. It represents 80-85% of the approximately 44,000 annual cases of thyroid cancer occurring in the United States [1]. Papillary thyroid carcinoma usually has an indolent course and remains localized. Approximately 5% of patients may develop metastatic cancer [2]. Pulmonary and bone metastases are most common. Patients with multi-organ involvement have a 15.3% five-year survival rate, while those with a single organ metastasis have a 77.6% five-year survival rate [3].

Metastatic thyroid cancers are treated with radioactive iodine (RAI), but approximately two-thirds of patients with distant metastases become RAI-refractory, meaning their tumors fail to take up RAI or progress despite uptake [3]. Prognosis in this setting is poor, and systemic options beyond multikinase and targeted inhibitors are limited [3].

Trophoblast cell-surface antigen 2 (Trop-2), a transmembrane glycoprotein encoded by the TACSTD2 gene, is overexpressed in many epithelial cancers, including breast cancer and papillary thyroid cancer [4]. Sacituzumab govitecan, an antibody-drug conjugate that delivers the topoisomerase I inhibitor SN-38 to Trop-2-expressing cells, is approved for metastatic triple-negative and hormone receptor-positive, HER2-negative breast cancer [5], but its activity in thyroid cancer has not been established.

The incidence of concomitant differentiated thyroid cancer and a secondary solid tumor is not well understood. An association between papillary thyroid carcinoma (PTC) and the subsequent development of non-thyroid malignancies has been reported, but the prevalence remains uncertain. Incidences reported by single-institution series lie in the range of 10-14% [6].

Here, we describe a patient with synchronous RAI-refractory metastatic thyroid cancer and hormone receptor-positive breast cancer who received sacituzumab govitecan, with an improvement in disease burden. This case suggests possible cross-tumor efficacy of a Trop-2-directed agent and supports further investigation of Trop-2 as a therapeutic target in RAI-refractory thyroid cancer.

## Case presentation

The patient is an 86-year-old female with a history significant for hormone receptor-positive, HER2-low (immunohistochemistry 1+) metastatic breast cancer. She was originally diagnosed with stage 1 disease treated with resection and adjuvant anastrozole for five years. She developed metastatic disease two years after completion of her adjuvant therapy. She received letrozole as initial endocrine therapy, followed by fulvestrant and ribociclib in the second line. She received capecitabine in the third line, with documented progression on each regimen.

Her history is complicated by metastatic papillary thyroid cancer. She was originally diagnosed with localized disease as systemic imaging did not reveal metastatic lesions. At diagnosis, her serum thyroglobulin (Tg) was 328 ng/mL (normal Tg value range: 20-25 ng/mL). She underwent total thyroidectomy, and pathology showed moderately differentiated papillary adenocarcinoma with local extension into skeletal muscle. Her thyroglobulin was 0.1 ng/mL, which was expected. Her case was presented at a multidisciplinary conference, and she was felt to be at high risk for recurrent disease. She then received a therapeutic dose (150 mCi) of radioactive iodine (RAI). Four years later, her Tg rose to 87 ng/mL. Imaging was negative for metastatic disease. A second dose of RAI was administered, and Tg became undetectable. Six months later, she developed a non-productive cough and the thyroglobulin level rose to 420 ng/mL. CT imaging revealed pulmonary nodules. After review with interventional radiology, a nodule was amenable to CT-guided biopsy, which was positive for thyroglobulin, thyroid transcription factor-1 (TTF-1), and paired box protein 8 (PAX8). The histology and associated markers confirmed metastatic papillary thyroid cancer (Figure 1).

**Figure 1:**
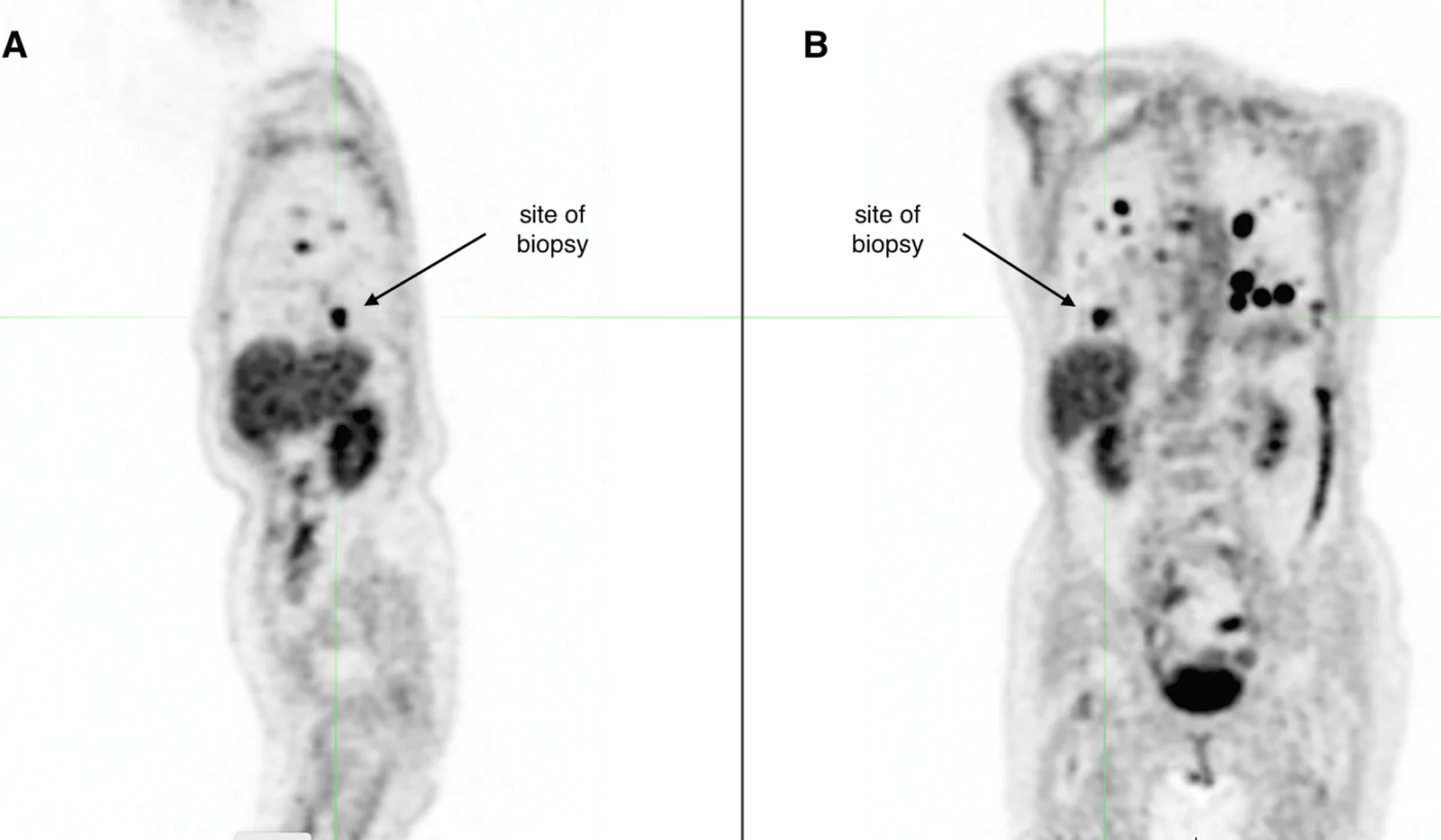
Positron emission tomography/Computed tomography (PET/CT) A: Sagittal cross-section revealing FDG-avid lung metastatic disease (black nodule). The arrow points to the biopsied mass. B: Coronal whole-body view demonstrating the FDG-avid metastatic disease. The arrow points to the nodule that was selected for biopsy due to anatomic location.

Next-generation sequencing (NGS) was performed on the tissue biopsy and identified a BRAF V600E mutation (Table 1).

**Table 1:** Next-Generation Sequencing: Genomic Variants BRAF p.V600E was identified as the primary actionable driver mutation. TERT promoter mutation and TP53 variants were classified as biologically relevant co-occurring alterations. GOF: gain of function; LOF: loss of function; VAF: variant allele fraction.

Gene	Alteration	Variant Type	Functional Effect	Variant Allele Fraction
Actionable				
BRAF	p.V600E	Missense variant	Gain of function (GOF)	3.3%
Biologically Relevant				
TERT	c.-124C>T	Promoter mutation	—	4.8%
TP53	p.R306Q	Splice region variant	Loss of function (LOF)	2.7%
TP53	p.R280T	Missense variant	Loss of function (LOF)	2.7%
Receptor Status				
HER2	—	—	Negative	—

Immunohistochemistry (IHC) for Trop-2 was performed on the biopsy sample and demonstrated moderate (2+) membranous staining (Figure 2).

**Figure 2:**
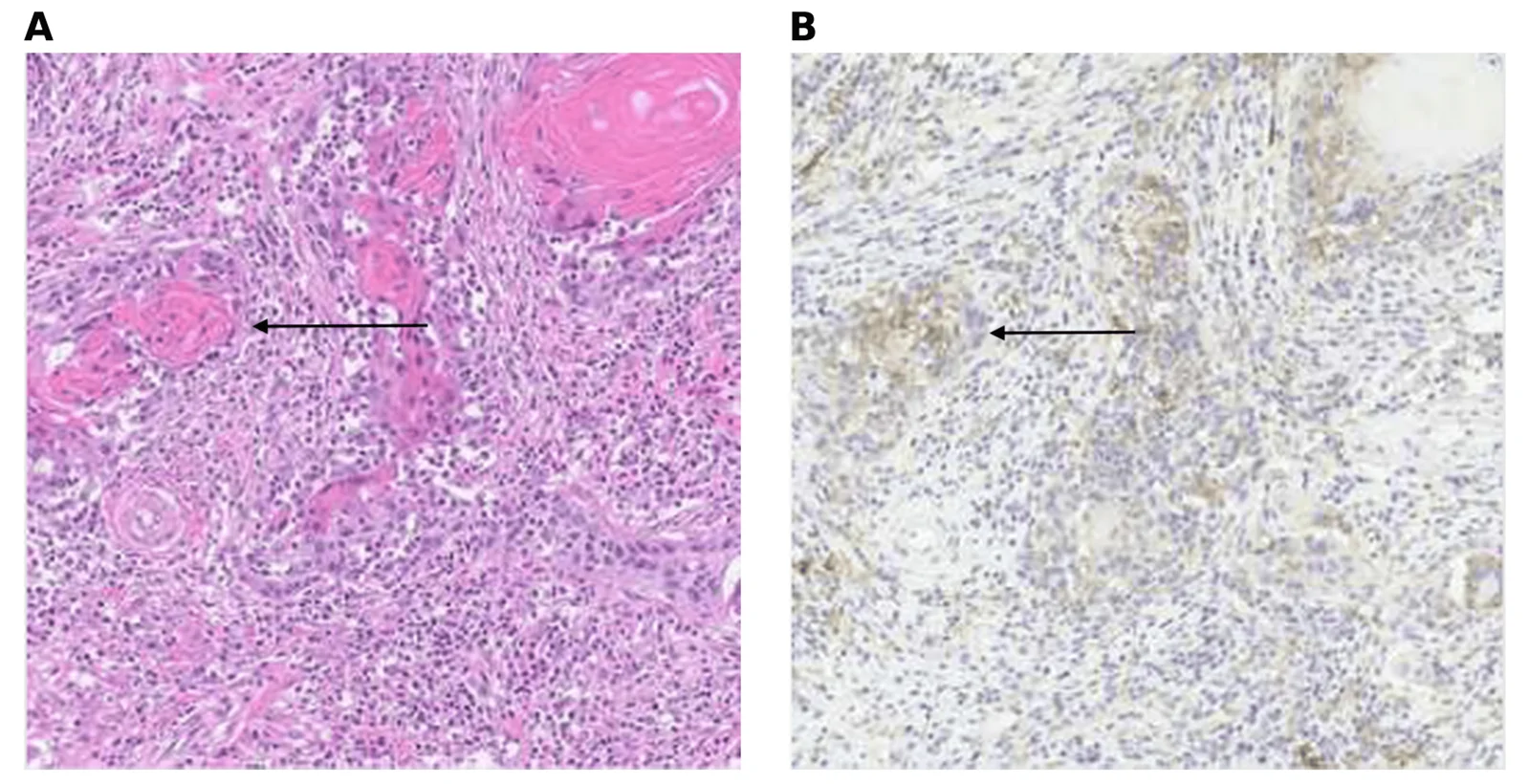
Lung biopsy tissue A: Lung biopsy with H/E stain. The arrow points to thyroid tumor infiltration in the lung parenchyma. B: Lung biopsy stained for Trop-2. The arrow points to immunohistochemistry positivity (brown color). This was read as moderate staining, 2+.

She was initiated on lenvatinib for the metastatic thyroid cancer as it is the standard of care for this clinical situation. It was discontinued shortly after due to poor tolerability, despite incremental dose reductions and treatment pauses. Sorafenib was given in the second line but was also discontinued after two months due to tolerability issues. Given her age and prior treatment tolerability concerns, BRAF-directed therapy was not pursued. Single-agent sacituzumab govitecan was subsequently initiated for management of her progressive metastatic breast cancer. At the time of treatment, it had no established indication for thyroid cancers. Prior to initiation, serum thyroglobulin was 709 ng/mL, CA 15-3 was 211 U/mL (normal 0-35 U/mL), and CA 27.29 was 190 U/mL (normal 0-37 U/mL).

Restaging after four cycles of treatment (approximately three months) demonstrated no new lesions and smaller lung masses. The index pulmonary lesions decreased from 3.1 cm to 1.5 cm and from 1.2 cm to 0.9 cm, representing a 44% reduction in the sum of longest diameters. No new lesions were identified. Concurrent serologic responses were observed across both malignancies: serum thyroglobulin declined from 709 ng/mL to 310 ng/mL (56% reduction), CA 15-3 declined from 211 U/mL to 75 U/mL (64% reduction), and CA 27.29 declined from 190 U/mL to 62 U/mL (67% reduction).

## Discussion

This case is notable for several reasons. The patient had two concurrent metastatic malignancies: hormone receptor-positive, HER2-low breast cancer and BRAF V600E-mutated papillary thyroid cancer. Both were refractory to prior therapy. Her thyroid cancer had progressed after two courses of RAI and was further complicated by intolerance to lenvatinib and sorafenib. Sacituzumab govitecan was initiated solely for her breast cancer. The subsequent partial radiographic response in all the pulmonary metastases, accompanied by a 56% decline in thyroglobulin and significant improvement of the breast tumor markers after four treatment cycles, raises the possibility that sacituzumab govitecan may have clinically meaningful activity against metastatic breast and papillary thyroid cancer through Trop-2 expression. As is standard practice, not every pulmonary lesion was biopsied, and the radiographic improvement was attributed to control of both malignancies.

Available treatments for advanced/metastatic differentiated thyroid cancers (DTC) primarily include the use of vascular endothelial growth factor receptor (VEGFR) inhibitors. Lenvatinib and sorafenib are considered the standard first-line treatment options in radioactive iodine-resistant patients, independent of mutation status [7]. For tumors positive for the BRAF V600E mutation, treatment approaches targeting BRAF, such as dabrafenib plus trametinib, can be used. There is significant variability in the clinical approach, and many physicians still opt for starting the VEGFR inhibitor treatment before resorting to BRAF-directed agents [8].

The prevalence of BRAF V600E mutation in patients with PTC has been reported in the 40-50% range [8]. Following the 2017 World Health Organization (WHO) reclassification of thyroid tumors, which excluded noninvasive follicular thyroid neoplasm with papillary-like nuclear features (NIFTP) from the PTC category, current series have reported significantly higher rates in the 78.8-86.8% range. NIFTP tumors preferentially harbor RAS rather than BRAF V600E mutations, and their exclusion from the PTC diagnostic category enriched the remaining cohort for BRAF-mutated cases. Other driver gene mutations account for an estimated 8-15.3% of cases [9]. Most patients ultimately acquire treatment resistance and experience disease progression, underscoring the need for novel therapeutic approaches in the later-line setting.

Sacituzumab govitecan is an antibody-drug conjugate (ADC). It is composed of a fully humanized anti-trophoblast cell-surface antigen 2 (Trop-2) monoclonal antibody linked to SN-38, the active metabolite of the topoisomerase I inhibitor irinotecan. Trop-2 is a transmembrane calcium signal transducer frequently overexpressed in epithelial malignancies and associated with dysregulated cell proliferation and tumor progression. Upon antibody binding and internalization, SN-38 is released within tumor cells and into the surrounding microenvironment, a phenomenon commonly referred to as the "bystander effect" [10].

In the phase III TROPiCS-02 trial, sacituzumab govitecan demonstrated improvement in progression-free and overall survival in pre-treated advanced hormone receptor-positive, HER2-negative breast cancer. This study included patients with no HER2 and HER2 low (IHC 1+ and IHC 2+/ fluorescent immunohybridization negative) expression [11]. To date, no approved indication exists for its use in differentiated thyroid malignancies.

Trop-2 expression has been documented in both breast and papillary thyroid carcinomas. This suggests that targeting a single mutation may provide clinical efficacy in both malignancies. Trop-2 expression in breast cancer correlates with response to sacituzumab govitecan, as demonstrated in TROPiCS-02 [12]. In this patient, thyroid cancer Trop-2 expression was confirmed by moderate (2+) membranous staining immunohistochemistry on the lung biopsy. This pattern is consistent with Trop-2 expression described in published series of papillary thyroid carcinomas [12].

Sacituzumab govitecan is felt to be effective by direct antibody-antigen binding and by a "bystander effect”. The bystander effect, whereby the toxin, SN-38, diffuses into the surrounding tumor microenvironment after release from Trop-2-expressing cells, potentiates cytotoxicity in areas of heterogeneous antigen expression [10]. This mechanism may be particularly relevant in the metastatic pulmonary setting, where tumor cell proximity could facilitate local drug distribution.

Recent preclinical studies have demonstrated that papillary thyroid cancers overexpress Trop-2. Single-center data have reported that up to 90% of papillary thyroid carcinomas exhibit a distinct membranous staining pattern for Trop-2, whereas no staining has been observed in benign thyroid lesions [12].

Sacituzumab Govitecan in Thyroid Cancers (SETHY; GETNE-T2318) represents the first clinical trial evaluating an antibody-drug conjugate in patients with advanced thyroid cancer [11]. This phase 2, open-label, prospective, multicohort, single-arm trial enrolls patients with advanced or metastatic RAI-refractory thyroid cancer, with sacituzumab govitecan dosed as per label as established in other malignancies. The trial is currently recruiting in Spain. The primary endpoint is objective response rate (ORR) assessed by serial imaging. Secondary endpoints include overall survival, progression-free survival, disease control rate, duration of response, safety, and quality of life [13]. Archival tumor specimens are collected at screening for retrospective centralized Trop-2 expression assessment and stored for subsequent correlative analyses. As of the date of publication, no interim efficacy data are available. A second phase 2 trial, SG-SGTC (NCT06923826), is also actively recruiting patients with RAI-refractory differentiated thyroid carcinoma, further reflecting growing clinical interest in Trop-2-directed therapy in this disease [14].

Concurrent response of two independent malignancies to a single systemic agent is uncommon. The magnitude of serologic response in this case, a 56% reduction in thyroglobulin and 64-67% reductions in breast tumor markers within three months, alongside a confirmed partial response by RECIST 1.1 in the pulmonary metastases, argues against spontaneous disease stabilization. The most straightforward explanation is that sacituzumab govitecan is exerting direct Trop-2-mediated cytotoxicity against both the breast and thyroid cancer clones, a mechanism supported by confirmed membranous Trop-2 expression on the thyroid metastasis biopsy. Systemic immune effects related to ADC-mediated tumor lysis may also contribute, though this remains speculative. The clinical trajectory in this case is most consistent with Trop-2-directed activity across both tumor histologies.

Patients with differentiated thyroid carcinoma have an increased prevalence of second primary malignancies [14]. This observation is likely attributed to expanded use of imaging that increases detection of additional malignancies. An increasing proportion of these malignancies harbor identifiable somatic alterations detectable by commercially available next-generation sequencing (NGS) assays. In a subset of patients, these alterations are shared across tumor types and may influence therapeutic decision-making when standard treatment strategies are limited. The development of mutation-directed therapies, including tyrosine kinase inhibitors and antibody-drug conjugates, has expanded available treatment options for select patients with dual primary cancers.

This report carries the inherent limitations of a single-patient observation. Causality cannot be established and the response may not be generalizable to the broader DTC population. The indolent clinical course of differentiated thyroid cancer introduces uncertainty. The documented serologic and radiographic progression prior to sacituzumab govitecan initiation, combined with confirmed Trop-2 expression on the thyroid metastasis, makes treatment-independent stabilization unlikely. Prospective data from the SETHY and SG-SGTC trials will be critical in determining whether the response observed in this case is reproducible.

## Conclusions

This heavily pretreated patient had two concurrent metastatic cancers: hormone receptor-positive, HER2-low breast cancer and BRAF V600E-mutated, RAI-refractory papillary thyroid cancer. Few systemic therapeutic options remained. Sacituzumab govitecan was given for her breast cancer. Restaging after four cycles showed a radiographic partial response in the pulmonary metastases, a 56% decline in thyroglobulin, and 64-67% reductions in breast tumor markers. Moderate (2+) membranous Trop-2 expression was confirmed on a thyroid metastasis. To our knowledge, this is the first reported response of papillary thyroid cancer to sacituzumab govitecan, and the first reported concurrent response of two primary malignancies to this agent. As a single observation, it cannot establish causality, but it is consistent with Trop-2-directed activity in both malignancies. Results from the SETHY and SG-SGTC trials will determine whether this activity is reproducible in RAI-refractory thyroid cancer.
